# Phosphuria in Diagnosis

**Published:** 1897-05

**Authors:** 


### PHOSPHURIA IN DIAGNOSIS.

Investigation of the phosphates is indispensable in some diagnoses, which would be very difficult without the knowledge thus obtained. The diagnosis of hysteria for instance, is settled when after the attack, the proportions of the phosphates are inverted and the weight of the phosphoric acid is normal or slightly diminished. On the other hand, epilepsy causes an increase of the phosphates and also of the phosphoric acid. In the same way ascertaining the conditions of the phosphates enables the differential diagnosis between tuberculosis and incipient chlorosis to be established. In cases of phosphuria the surgeon should always reserve his prognosis even in the case of wounds and fractures and as well when intervention is being considered.—*Nord Medical.*

